# Expanding the Diversity of Imaging-Based RNAi Screen Applications Using Cell Spot Microarrays

**DOI:** 10.3390/microarrays2020097

**Published:** 2013-04-11

**Authors:** Juha K. Rantala, Sunjong Kwon, James Korkola, Joe W. Gray

**Affiliations:** Department of Biomedical Engineering and Knight Cancer Institute, Oregon Health and Science University, Portland, OR 97239, USA; E-Mails: kwons@ohsu.edu (S.K.); korkola@ohsu.edu (J.K.); grayjo@ohsu.edu (J.W.G.)

**Keywords:** RNA interference, high-throughput screening, cell spot microarrays, image cytometry, quantitative immunofluorescence assays

## Abstract

Over the past decade, great strides have been made in identifying gene aberrations and deregulated pathways that are associated with specific disease states. These association studies guide experimental studies aimed at identifying the aberrant genes and networks that cause the disease states. This requires functional manipulation of these genes and networks in laboratory models of normal and diseased cells. One approach is to assess molecular and biological responses to high-throughput RNA interference (RNAi)-induced gene knockdown. These responses can be revealed by immunofluorescent staining for a molecular or cellular process of interest and quantified using fluorescence image analysis. These applications are typically performed in multiwell format, but are limited by high reagent costs and long plate processing times. These limitations can be mitigated by analyzing cells grown in cell spot microarray (CSMA) format. CSMAs are produced by growing cells on small (~200 μm diameter) spots with each spot carrying an siRNA with transfection reagent. The spacing between spots is only a few hundred micrometers, thus thousands of cell spots can be arranged on a single cell culture surface. These high-density cell cultures can be immunofluorescently stained with minimal reagent consumption and analyzed quickly using automated fluorescence microscopy platforms. This review covers basic aspects of imaging-based CSMA technology, describes a wide range of immunofluorescence assays that have already been implemented successfully for CSMA screening and suggests future directions for advanced RNAi screening experiments.

## 1. Introduction

**Figure 1:**
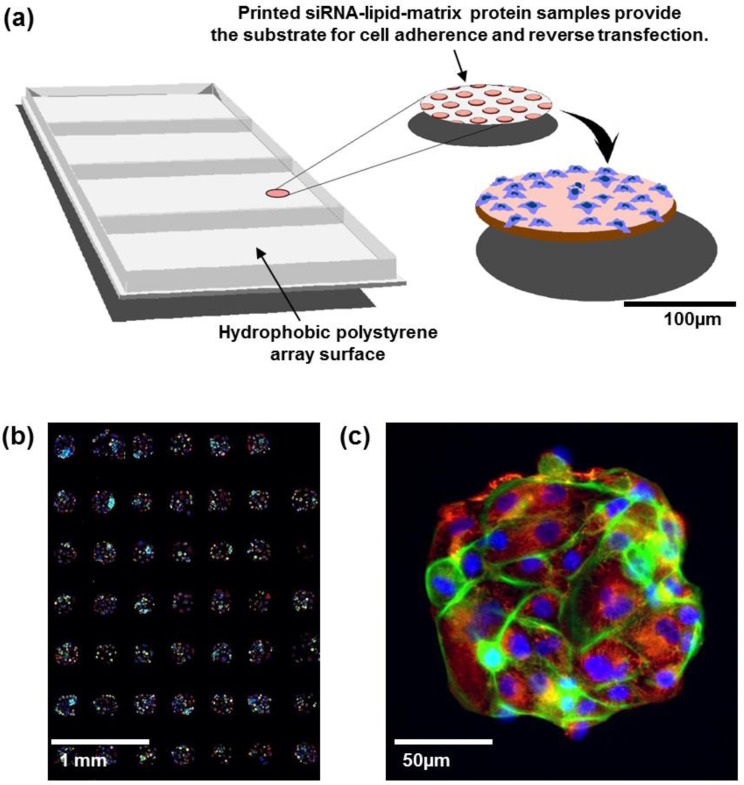
(**a**) Cell spot microarrays (CSMAs) are produced by spotting siRNA samples mixed with transfection lipids and extra-cellular matrix proteins on a hydrophobic polystyrene surface in microplate sized vessels. This enables production of high density cell transfection microarrays with up to 1,000 siRNA samples/cm^2^. The slides are coated so that the cells adhere only to the spots containing the siRNAs. Transfer of the siRNAs to the cells occurs by reverse transfection during growth for a selected time, typically 48 to 72 h. (**b**) Multicolor image of immunofluorescently stained human kidney tumor cells following growth in CSMA format. The diameter of a cell spot is approximately 200 μm. (**c**) High resolution image of cells growing on one cell spot stained for DNA (blue), F-Actin (green) and beta-tubulin (red).

International genomic studies are revealing a growing number of genomic aberrations and aberrant pathways that are postulated to be important in a wide range of human diseases including cancer. The number of candidate “cancer genes” emerging from these efforts is especially daunting. However, the definitive functions of these aberrant genes and pathways must be established by experimental manipulation in laboratory models. Manipulation of gene expression levels using inhibitory RNAs (RNAi) is a key technique for this purpose. Large-scale nucleic acid synthesis techniques now enable convenient and low cost synthesis of thousands of RNAis so that assessment of the effects of manipulating the expression levels of thousands of genes is possible. Initial efforts in RNAi screening in mammalian cells were accomplished using automated strategies in which the effects of RNAi knockdown were assessed in multiwell format (usually 96- or 384-well plates). This required substantial laboratory instrumentation and automation, while the costs of RNAi reagent libraries were high. A strategy to miniaturize this process was first suggested by Sabatini *et al.* [1] and subsequently demonstrated with both siRNAs and shRNAs [2,3,4,5] before fully developed by Rantala *et al.* [6]. In this process, individual RNAi oligonucleotides are printed in ~200 μm diameter spots separated by a few hundred micrometers (Figure 1(a)). Each spot carries cell adherence promoting matrix proteins allowing spatially confined array patterning with cells growing only on the spots. Each spot can host up to a few hundred cells and contains a lipid transfection agent so that cells auto-transfect as they grow. This miniaturized platform provides an economical and robust alternative to multiwell screening systems for systematic assessment of gene function *in vitro*. In a typical experiment, siRNA-lipid microarrays are covered with adherent cells in a culture medium for reverse transfection-mediated uptake of the siRNA [6] or micro-RNA [7] reagents from the spatially confined array spots. siRNAs usually are arrayed in triplicate in order to enable assessment of experimental reproducibility. Spot densities of ~1,000/cm^2^ are routinely achieved on this platform so that responses to thousands of siRNAs can be robustly assessed in a single culture [6]. The well-less and miniature format of the CSMA platform allows cells to be immunofluorescently stained for specific molecular response endpoints (e.g., molecular events associated with proliferation, apoptosis, differentiation status, senescence, *etc*.) much as one would stain cells grown on a coverslip (Figure 1(b,c)). Alternately, cells can be genetically engineered to express fluorescent response reporter constructs for time-resolved analyses [6,8].

The quantitative assessment of the impact of high-throughput RNAi knockdown on immunofluorescently stained molecular features—the subject of the present article—has already been applied in several cell biological studies, and the potential of the approach is only beginning to be realized. Early applications include analysis of the impact of specific RNAi-induced knockdowns on cellular abundance of protein complexes [9], regulatory pathways [10,11], and changes in the spatial distribution of target proteins [12]. We describe here experiments using siRNAs as RNAi reagents, but the platform appears readily extensible to assessment of effects of shRNAs [13], miRNAs [7] and cDNAs [1]. The applications described herein illustrate how the CSMA platform can be used for efficient assessment of the roles that specific molecular entities or genomic aberrations play in many aspects of cancer pathophysiology. The ability to assess the impact of RNAi knockdown on endpoints such as differentiation, DNA repair activity, senescence and motility is a particular strength of the platform. The advantages of the platform relative to multiwell analysis approaches are the high analysis density which allows detailed analyses of responses to thousands of RNAis at low immunochemical reagent and cell culture cost and relatively high analysis speed. The disadvantages of the platform are the relatively small numbers of cells interrogated on each spot and the possibility that cells on one spot may be affected by a small amount of molecule secretion from cells on adjacent spots. Printing replicate spots to increase the number of cells analyzed can mitigate the former disadvantage and the latter can be mitigated by randomly positioning replicate spots across the culture surface so that each replicate is in close proximity to a different collection of other RNAi-perturbed cell spots. A typical experiment in which each RNAi is printed in triplicate provides analyses of sufficient cells to enable detection of RNAi effects that differ from the control by less than 10%. RNAi against genes like AURKB, CDK1, INCENP, KIF11 and PLK1 for which responses are well established are usually included as positive controls [6,8,14] to confirm that the RNAi transfection efficiency is high. This is especially important when working with cell types that have not been previously analyzed using the CSMA platform [6].

The advantages of assessing RNAi-induced changes using image cytometry following immunofluorescence staining—whether in CSMAs or in multiwell cultures—are substantial compared to strategies that assess bulk changes in cell number or metabolic activity (e.g., using the CellTiterGlow or MTT assays; [15]) or that identify RNAi effects following bulk transfection (e.g., by assessing loss of cells carrying specific RNAis). Specifically, imaging allows for the following: (a) Quantitative measurement of the cellular abundance of specific target molecules for which a fluorescence reporter can be developed. These studies take advantage of a growing number of antibodies, aptamers or other affinity ligands that bind with high affinity and specificity to proteins or post-translationally modified variants that comprise regulatory networks. The availability of fluorescent reporter constructs that level the expression levels of specific proteins further increases the information that can be obtained; (b) Assessment of the intracellular distributions of the proteins or organelles of interest. This enables assessment endpoints such as the number of discrete DNA repair foci (a measure of DNA repair activity), assessment of the fraction of cells incorporating EdU, mitochrondrial morphology and assessment of mitotic apparatus shape; (c) Analysis of molecular proximity through the use of Förster resonance energy transfer assays (FRET) or antibody-based proximity ligation assays (PLA); (d) Assessment of molecular response heterogeneity between the cells in a single cell spot—for example, induced by cell–cell proximity and/or transient differentiation; (e) Multiplex analysis of multiple molecular and biological response endpoints. For example, multicolor analysis allows assessment of how RNAi manipulation changes the relationship between molecular pathway components (e.g., PI3K and MAPK pathway activities), and biological endpoints such as EdU incorporation or cell cycle distribution, motility, differentiation status and cell death [7,10,11,16,17]; (f) Assessment of the impact of RNAi knockdown on responses to chemical, biological or microenvironmental perturbations. The platform is especially useful in identifying genes and pathways that influence responses to anticancer agents. 

In the following sections, we describe fundamental aspects of image cytometry-based RNAi screening applications on CSMAs and review some of the basic methods. We also discuss in detail the custom assays established in our laboratory for analysis of quantitative cancer cell phenotypes. Finally, we conclude with an assessment of future developments of imaging-based RNAi analyses.

## 2. Experimental Section

### 2.1. Cells and Cell Culture

All cell lines grown on CSMAs were cultured according to the protocols recommended for the cell line. Primary kidney tumor cells were isolated from fresh patient surgical specimens obtained under an Institutional Review Board-approved protocol at Oregon Health and Science University (OHSU). These primary kidney cells, along with BT20, HCC1569, HCC1954, MDA-MB-468, U-2OS, KFr13, VCaP, and 22RV1 cells (ATCC, Manassos, VA, USA) were grown in RPMI-1640 (Gibco, Life Technologies, Grand Island, NY, USA) supplemented with 10% FBS, 10 µg/mL penicillin and streptomycin and 2 mM l-glutamine. HaCat, MFC7 and MDA-MB-231 cells were grown in DMEM (Gibco) supplemented with 10% FBS, 10 µg/mL penicillin and streptomycin and 2 mM L-glutamine. RWPE-1 (ATCC) cells were grown in Keratinocyte Serum Free Medium (K-SFM, Gibco) supplemented with 0.05 mg/mL BPE (bovine pituitary extract) and 5 ng/mL EGF. SKBR3 cells (ATCC) were grown in McCoy’s 5A medium (Gibco) supplemented with 10% FBS, 10 µg/mL penicillin and streptomycin, and 2 mM l-glutamine.

### 2.2. Preparation of Cell Spot Microarrays

Transfections with siRNAs and cell culture on the CSMAs were carried out as described previously [6]. siRNAs against PLK1 and AURKB purchased from Qiagen as experimentally verified oligos were used for transfection validation experiments (PLK1 #A SI02223837, #B SI02223844; AURKB #A SI02622032, #B SI02622039). Briefly, the siRNAs and siLentFect (Bio-Rad) transfection reagent for array printing were prepared by mixing the lipid–siRNA samples with cold growth factor-reduced Matrigel (BD Biosciences, Bedford, MA, USA)—OptiMEM I (Gibco) solution resulting in final siRNA printing concentrations of 2.5 µM and 15% Matrigel. These solutions were printed as 200 µm diameter spots on the bottom of the wells of polystyrene microplates (typically 4 to 8 wells per plate, Nunc Brand, Roskilde, Denmark). The siRNA–Matrigel spots were allowed to polymerize for 30 min at room temperature and then stored at room temperature, desiccated and protected from light [6]. Arrays were stored for several weeks before use under these conditions. Approximately 2 × 10^6^ cells in 4.5 mL of growth medium were added to each array well (4-well plates) and allowed to adhere at +37 °C for 5–15 min. Cells were dispersed with non-trypsin cell detachment reagent HyQtase (HyClone, South Logan, UT, USA) prior to seeding on the CSMAs since this dispersal method enabled rapid adhesion to the array spots. Non-adherent cells were washed off and 4.5 mL of fresh medium was added per array well. siRNA transfer to the adherent cells took place during growth periods ranging from 48 to 144 h prior to staining and imaging.

### 2.3. Antibody Staining Procedure

Cells transfected with siRNAs during growth on CSMAs were immunofluorescently stained according to the following protocol. First the culture medium was aspirated carefully from each array well and the cells were fixed with 2% paraformaldehyde (Sigma-Aldrich, St. Louis, MO, USA) in PBS for 15 min at room temperature. Cells were then rinsed once briefly with 50 mM NH_4_Cl to quench any remainder of the paraformaldehyde fixative and washed 5 min with PBS. Cells were permeabilized with 0.3% Triton-X100 in PBS for 15 min at room temperature, washed once with PBS and blocked with 2% filtered BSA in PBS for 60 min at room temperature. After blocking, the arrays were washed 2 × 5 min with PBS, rinsed briefly with distilled H_2_O and air-dried. Array areas were inscribed with a hydrophobic border using a PAP-pen (Sigma-Aldrich) to reduce the amount of antibody used during staining. Cells were rinsed with 0.05% PBS-Tween 20 and stained with primary antibody in 2% BSA-PBS (100 μL per 20 × 20 mm array surface) for 1 h at room temperature or overnight at 4 °C. These arrays were washed 2 × 5 min with PBS and for 5 min with 0.05% PBS-Tween 20 and then stained with 100 μL of diluted Alexa fluorochrome-conjugated (Life Technologies) secondary antibodies. Secondary antibody incubation and parallel DAPI counterstaining was performed for 1 h at room temperature, followed by washing as described for primary antibodies. The stained arrays were then rinsed with distilled H_2_O, air-dried and stored for imaging. The cells were rehydrated for imaging by covering the arrays with PBS or by mounting under a coverslip using ProLong Gold anti-fade reagent (Life Technologies).

### 2.4. RNA Immuno-FISH Procedure

Cells grown on CSMAs were fixed in 4% formaldehyde in PBS for 10 min, permeabilized with 70% ethanol at 4 °C for 1 h, and prehybridized with wash buffer (2× SSC, 10% formamide) at room temperature for 10 min. Cells were then incubated in parallel with cyclin D1 antisense-oligonucleotide probe-sets labeled with Cy-5 and anti-Ki67 antibody (1:300, Abcam, Cambridge, MA, USA) in hybridization solution (10% Dextran sulfate, 2× SSC, 10% formamide) at 37 °C overnight in a dark/humid chamber, and incubated with wash buffer at 37 °C for 30 min twice. Secondary antibody incubation (Alexa488-conjugated goat anti-rabbit antibody in the hybridization solution) was carried out at room temperature for 1 h, followed by incubation with wash buffer for 30 min, with DAPI for nuclear staining, and with 2× SSC. Cells on arrays were mounted in Prolong Gold anti-fade reagent (Life Technologies). Probe sets for CCND1 mRNA detection were designed using a Stellaris™ Probe Designer version 1.0 [18]. They were composed of 48 different 20 mer DNA oligonucleotides, each complementary to a different region of CCND1 mRNA, targeting sequences with 45% GC content, separating at least two bases between oligonucleotides. Images of 0.2 µm optical sections were acquired using Deltavision CoreDV Automated Widefield microscopy (Applied Precision™, 60× objective, NA = 1.42) with a Nikon Coolsnap ES2 HQ camera. These images were processed with deconvolution software to subtract blurred lights or to reassign them back to sources, and reconstructed into 3D image using IMARIS™ software (Bitplane, South Windsor, CT, USA).

### 2.5. Antibody-Based Proximity Ligation Assay for *in situ* Protein–Protein Interaction Analysis

After transfection, cells grown on CSMA were fixed and stained according to the manufacturer instructions for the DuoLink II PLA kit (Olink, Uppsala, Sweden) with a minor change in PLA probe dilution. The primary antibodies were diluted 1:200 in 2% BSA-PBS and incubated overnight at 4 °C (ITGB1; Abcam 12G10, ITGA2; Millipore AB1936). The PLA probe antibodies were diluted 1:20 in 2% BSA-PBS supplemented with the PLA blocking concentrate and incubated for 2 h at 37 °C. The array surfaces were circumscribed with a hydrophobic border drawn with a PAP-pen (Sigma-Aldrich) to minimize antibody and PLA detection reagent consumption. Cells were counterstained for filamentous actin using fluorescently labeled phalloidin (Alexa488, Life Technologies) and DNA (DAPI). Images for each spot in the CSMAs were acquired automatically using an Olympus scan^R imager and image analysis software (Olympus-SIS, Münster, Germany) was used to quantify PLA levels in individual cells.

### 2.6. Western Blot Analysis

Western blot analysis of total cell lysates prepared from cells transfected on CSMAs with 384 replicate spots of a single control or targeting siRNA were fractionated on SDS-polyacrylamide gels and transferred to nitrocellulose membranes (Whatman Inc., Kent, UK). The membranes were blocked against non-specific binding using 5% skim milk. Membranes were probed with primary antibodies (PLK1, Abcam; AURKB, Abcam) overnight at 4 °C. Equal loading was confirmed by probing the same filter with a specific antibody for β-tubulin or β-actin (1:5,000, Abcam). Signals were revealed with horseradish peroxidase-coupled secondary anti-mouse or anti-rabbit IgG antibodies (1:1,000; Sigma-Aldrich).

### 2.7. Imaging and Analysis

Array imaging was performed using an Olympus scan^R integrated imager and image analysis suite (Olympus-SIS, Münster, Germany) equipped with a Hamamatsu ORCA-R^2^ CCD digital camera (Hamamatsu Photonics K.K., Tokyo, Japan). The Olympus scan^R system is an inverted microscope designed for fully automated image acquisition of biological samples in high-density sample platforms such as CSMA plus image analysis algorithms for feature quantification. Each spot on a CSMA was imaged individually with a 20× LUCPLFLN NA 0.40 objective using specific filter sets for DAPI, Alexa488, Alexa568 and Alexa647 dyes (Semrock, Inc., Rochester, NY, USA). The scan^R image analysis software suite was also used for quantitative analysis of image features. Analysis capabilities included cell/particle counting, protein expression analysis with immunofluorescence quantitation, subcellular particle quantitation assays, cell cycle analysis, and protein localization and co-localization assays. Image features quantified for cell populations using the scan^R software were further analyzed using FCS Express 3.0 software (De Novo Software, Los Angeles, CA, USA). The effects of each siRNA knockdown on each specific response endpoint were assessed by comparing image parameters (e.g., total fluorescence intensity per cell, fraction of cells incorporating EdU or number of segmented spots per cell) with comparable image parameters measured for cells transfected with a non-targeting scrambled control siRNA.

## 3. Results and Discussion

### 3.1. Image Cytometry of Cell Spot Microarrays

**Figure 2:**
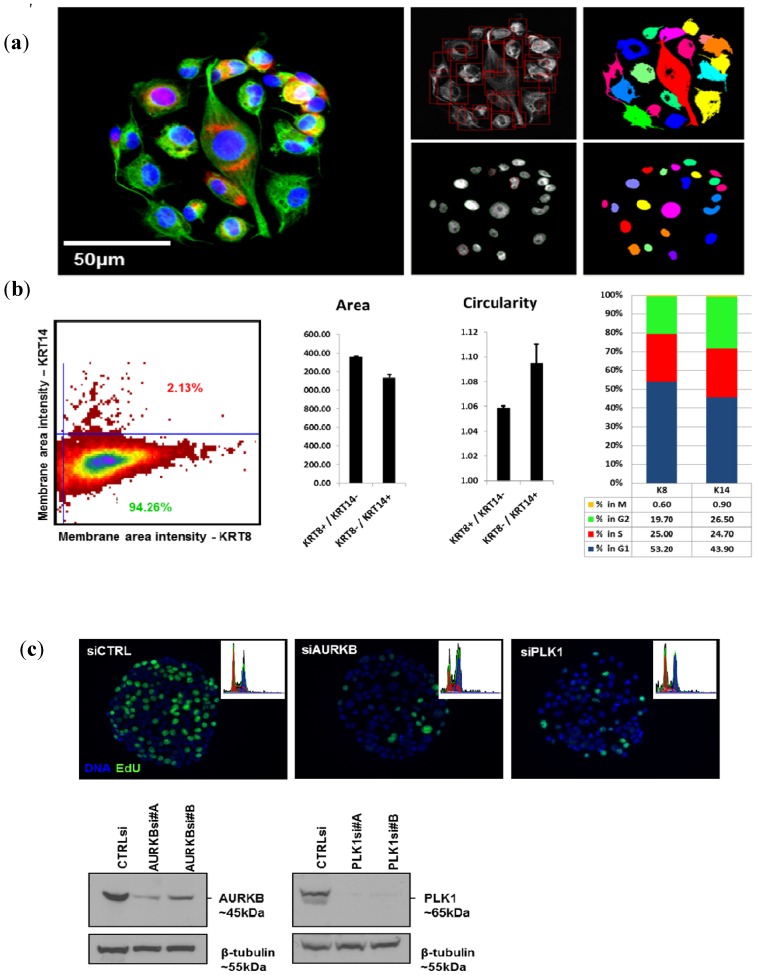
Examples of quantitative image cytometry on CSMAs. (**a**) BT-20 breast cancer cells stained for DNA (blue), cytokeratin-8 (green) and -14 (red). Panels on the right show segmentation of the image according to DAPI and cytokeratin extent. (**b**) Identification of a subpopulation of KRT14 positive cells comprising 2% of the KRT8 positive parental BT-20 cell population (left panel). The panels on the right show that KRT14 positive cells had smaller, less circular nuclei and a different distribution of cells in the G1 and G1 phases of the cell cycle compared to KRT8 positive cells. (**c**) Assessment of the effects on cell cycle traverse of siRNA knockdown of AURKB and PLK1 in KFr13 ovarian cancer cells after 48 h. Panel c shows images of DAPI and EdU incorporation 48 h after growth on cell spots carrying a scrambled siRNA and siRNAs against PLK1 and AURKB. Insets show DNA distributions calculated from nuclear DAPI intensity measurements. These analyses show the decrease in proliferation and the increase in frequency of polylobed (siAURKB) or mitotic (siPLK1) cells induced by siRNA knockdown. The lower panels show levels of AURKB and PLK1 proteins after growth on CSMAs carrying siRNAs against AURKB and PLK1, respectively.

Figure 2(a) illustrates the results obtained using an Olympus scan^R imager for analysis of cells grown in CSMA format. The scan^R software was used to define cell based on the extent of immunofluorescently stained cytokeratin and nuclear boundaries defined based on the extent of DAPI-stained DNA. Segmentation of secondary objects within the primary objects was then performed to define and quantify subcellular features. These measurements of subcellular and cellular shape features in adherent cells are the unique provenance of image cytometry since they cannot be readily assessed using flow cytometry. Figure 2(a), for example, shows the use of combinations of cell morphology parameters and cell differentiation markers to identify biologically distinct subpopulations within a cell culture environment that is often presumed to be homogeneous. In this example, BT-20 human breast cancer cells seeded on CSMAs were stained for basal cell lineage markers cytokeratin-8 and -14 and the sub-populations staining positive for one but not the other marker (94.26% KRT8+, 2.13% KRT14+; Figure 2(b)) were analyzed separately for nuclear size and cell cycle distribution. This analysis, which highlights the multiplexing capabilities of the image cytometry assays, indicated these two cell populations differed in both features. The KRT8-/KRT14+ cells within the parental population had consistently smaller, less circular nuclei and an increased fraction of cells in the G_2_-M-phase of the cell cycle (Figure 2(b)).

Another example of the robustness of the image cytometry is its ability to assess cell cycle features. These analyses can be performed simply on the basis of a single DNA dye such as DAPI, PI or Hoechst in combination with nuclear size and shape measures. Measurements of the total nuclear fluorescence in proportion to the nuclear size of cells generates scatter plots of DNA content to nuclear area ratios that can be gated to accurately quantify percentages of cells in definite G_1_-, S- and G_2_-phases of cell cycle. The mean pixel intensity corresponding to chromatin condensation and the nuclear area ratio enables mitotic and late anaphase cells to be distinguished from G_2_ and G_1_ cells, respectively [9]. In addition, apoptotic cells can be separated from mitotic cells without use of any specific markers based on the size and circularity of the objects. Figure 2(c) shows the use of these features to assess responses to RNAi knockdown of well-established cell cycle regulating proteins, AURKB and PLK1. This type of cell cycle analysis may be performed directly from DNA measurements, leaving other available fluorescence channels available for assessment of additional markers of interest. As an example, assaying EdU (5-ethynyl-2'-deoxyuridine, Life Technologies) incorporation (Figure 2(c)) or detection of Histone-H3 phosphorylation [14] can be used to further fine-tune analyses of specified cell cycle phases. This ability to measure shape and quantity of multiple stained features in individual cells enabled by image cytometry allows coordinate analysis of cell cycle endpoints and the expression levels of the proteins that control cell cycle traverse. 

### 3.2. Surrogate Markers for Analysis of Quantitative Cancer Cell Phenotypes

High-throughput RNAi screening has become a major tool for identification of molecular functions and genomic aberrations that play a role in cancer pathophysiology and for identification of novel candidate therapeutic targets. These studies have been, and continue to be, important in categorizing general molecular networks crucial for cell survival and proliferation. However, assessment of cell viability provides little information about the detailed roles that the interrogated target genes may play in the other important aspects of cancer cell physiology. Decades of research have defined an atlas of cellular functions that go awry in cancer development and progression and manifest in the cancerous cells as changes of molecular and structural phenotypes. These key aberrant cellular features of cancer have been designated collectively as “hallmarks of cancer” [19].

**Figure 3:**
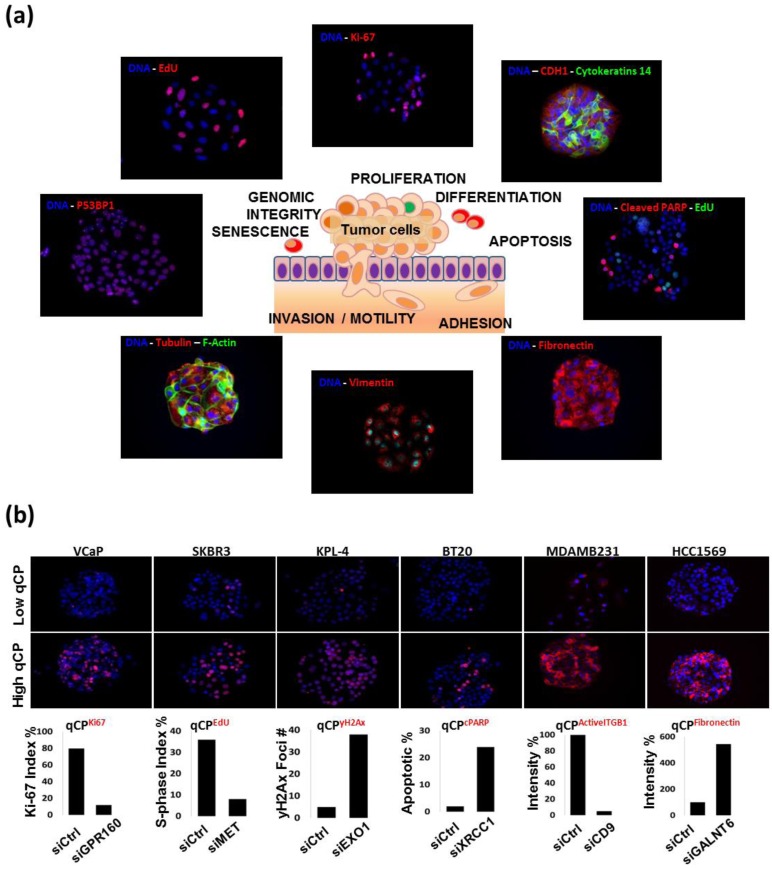
(**a**) A gallery of representative images showing immunofluorescent staining patterns that can be analyzed as quantitative cancer cell phenotypes (qCP). These assays assess aspects of cell proliferation, differentiation, apoptosis, cell adhesion/motility, senescence and genomic integrity. Each marker is indicated on the images with text in the corresponding color. The complete list of the validated qCP detection reagents is listed in Table S1. (**b**) Assessment of changes in qCPs induced by siRNAs targeting genes known to produce change in specific cancer hallmarks. qCPs were measured after growth on targeting or control siRNAs for multiple cell lines. The cell lines, targeting siRNAs and assessed qCPs are indicated in the figure.

We have now developed immunofluorescent staining and quantitative image analysis procedures for many of the key molecular features associated with the cancer hallmarks to facilitate use of RNAi screening for in-depth biological discovery in the context of cancer research. Measures of RNAi-induced changes in these features—designated herein as quantitative cancer phenotypes (qCPs)—enables accurate identification of aberrant genes or networks that are causal for many aspects of cancer. This approach assesses many more aspects of cancer physiology than are routinely assessed in screening strategies that are sensitive only to events that alter proliferation or immortalization. We are now applying this approach to systematic assessment of oncogenic events, signal transduction programs and associated deregulated gene networks that are postulated to contribute to the hallmarks of cancer by the Cancer Genome Atlas (TCGA) [20] and related international genomics efforts. Our current system measures qCPs that report on proliferation, apoptosis, senescence, differentiation, DNA replication and repair, and motility (Figure 3(a)). The cell proliferation qCP quantifies the fraction of cells incorporating a nucleotide analog (EdU or BrdU) or showing high level staining for Ki-67 [6]. The apoptosis qCP reports the level of staining for cleaved PARP [7] or members of the caspase protein family. The qCP for cell senescence reports the intensity of staining for trimethylation of histone 3 lysine 9 (H3K9me^3^), an established marker for heterochromatin formation. Epithelial-to-mesenchymal transition is one of the fundamental cellular phenotypes associated with the course of cancer pathogenesis. qCPs for differentiation status report the intensity of staining for differentiation associated proteins including Beta-Catenin, Cytokeratins-8/14/19, E-Cadherin, EpCAM, Fibronectin and Vimentin [21]. Loss of genomic integrity and aberrant DNA repair is characteristic of virtually all human cancers [22]. The qCP for DNA damage repair (DDR) reports the number of detect P53BP1 or gamma-H2Ax foci that are associated with DNA double-strand breaks [9,21]. Figure 3(a) shows a gallery of CSMA images of cells stained with antibodies that report on each of these cancer hallmarks. The complete description of the qCP marker antibodies that we now use for image-based analysis of qCPs is provided in Table S1. The utility of these assays for large-scale CSMA screens has been confirmed in our previous and on-going work [6,10,11,16,17]. Representative examples of siRNA-induced changes in qCPs are shown in Figure 3(b). Our panel of assays is not intended to be a definite list of assays for quantitative cancer phenotype assessment. Rather, it is a guiding collection setting the basis for future development of additional image cytometry assays for RNAi screening experiments that will contribute to the systematic assessment of the roles specific aberrations play in cancer pathophysiology.

### 3.3. Next-Generation RNAi Screen Readouts—Immuno-FISH on CSMAs

**Figure 4:**
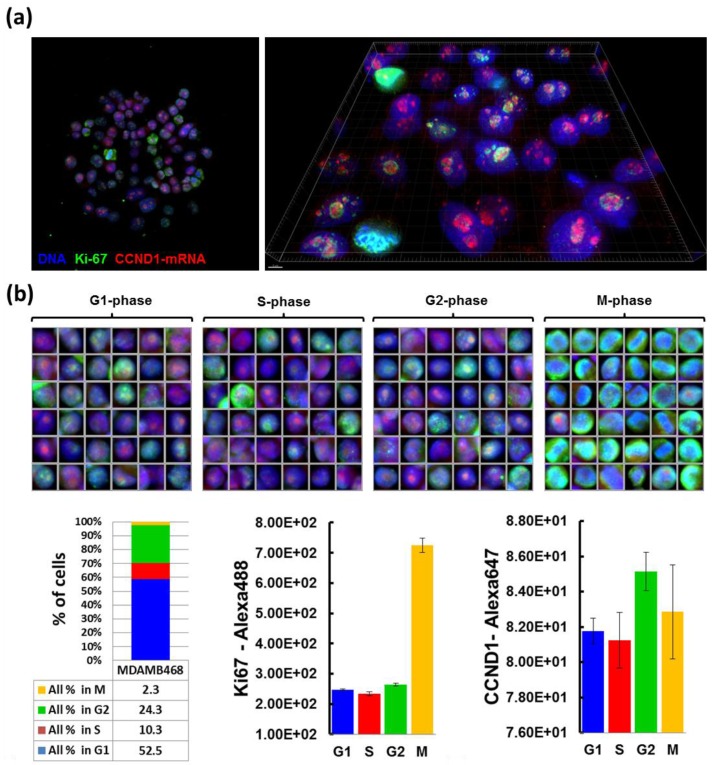
(**a**) Quantitative RNA immuno-FISH on CSMAs. MDAMB468 breast cancer cells grown on a CSMA were immunostained in for Ki-67 (green) and for CCND1 using RNA-FISH (red). Nuclei were counterstained with DAPI (blue). The left panel shows a scan^R image at 20× magnification. The right panel shows a high resolution widefield microscopic image of the same field. Red punctate dots with intense CCND1 mRNA staining in nuclei indicate localization of the CCND1 messenger to the nucleoli. Scale bar 5 μm. (**b**) Images of individual cells showing nuclear CCND1 RNA levels and Ki-67 staining (upper panel). The cells are organized according to cell cycle phase as determined from quantitative measurements of nuclear DAPI fluorescence. The lower panel shows quantitative analyses of the images in panel b. These analyses show that mitotic cells have the highest mean nuclear area signal for Ki-67 while cells in G_2_-phase have the highest nuclear intensity for CCND1 mRNA (lower right panels). Error bars indicate the variance present that resulted from analysis of 1,686 cells with 885 (52.5%) in G_1_, 173 (10.3) in S, 409 (24.3%) in G_2_ and 39 (2.3%) in M-phase (lower left panel).

The miniature scale of the CSMA platform also facilitates development of other molecular state assays such as Fluorescence *in situ* Hybridization (FISH) for genome number (DNA-FISH) [23], or abundance and cellular location of mRNA transcripts (RNA-FISH). These approaches rely on the *in situ* detection of specific DNA or RNA sequences in fixed cell samples using probe oligonucleotides that are complementary to the specific sequence(s) of interest. We tested the performance of RNA-FISH for quantification of RNA levels altered during RNAi screening by assessing CCND1 (Cyclin D1) mRNA levels. We use the Stellaris FISH method (Biosearch Technologies) in which detection probes are comprised of multiple short, fluorescently labeled oligonucleotides targeting one mRNA transcript [24]. The high signal amplification achieved in this procedure produces signals that can be readily detected and quantified during imaging. The Stellaris FISH technique does not require denaturation of the samples with heat so the staining procedure can be combined with other detection techniques such as immunofluorescent staining. Figure 4(a) shows a prototypic example in which we combined RNA-FISH detection of CCND1 transcripts with immunofluorescent staining for Ki-67. Dual-stained MDA-MB-468 breast cancer cells were imaged with the Olympus scan^R and a Deltavision CoreDV widefield microscope. These studies showed that CCND1 mRNA was located predominantly in the nuclei, and more specifically to nucleoli. We assessed signal intensities of the nuclear CCND1 mRNA and Ki-67 immunofluorescence staining in cells assigned to the G1-, S-, G2- and M-phases of the cell cycle phases based on DAPI fluorescence intensity and spatial distribution. In this example, nuclear staining for Ki-67 was highest in mitotic cells as previously shown [6] and nuclear CCND1 staining intensity was modestly higher in G_2_ cells in comparison to the other groups (Figure 4(b)).

We anticipate that use of immuno-FISH as described coupled with high-throughput RNAi screens will enable coordinate analysis of the impact RNAi knockdown on both transcriptional and translational regulation of target genes and proteins. This will allow discrimination between RNAi effects that directly alter expression of the target genes and those that affect post-translational mechanisms regulating the protein functions. 

### 3.4. Next-Generation RNAi Screen Readouts—Analysis of Endogenous Protein Interactions *in situ*

**Figure 5:**
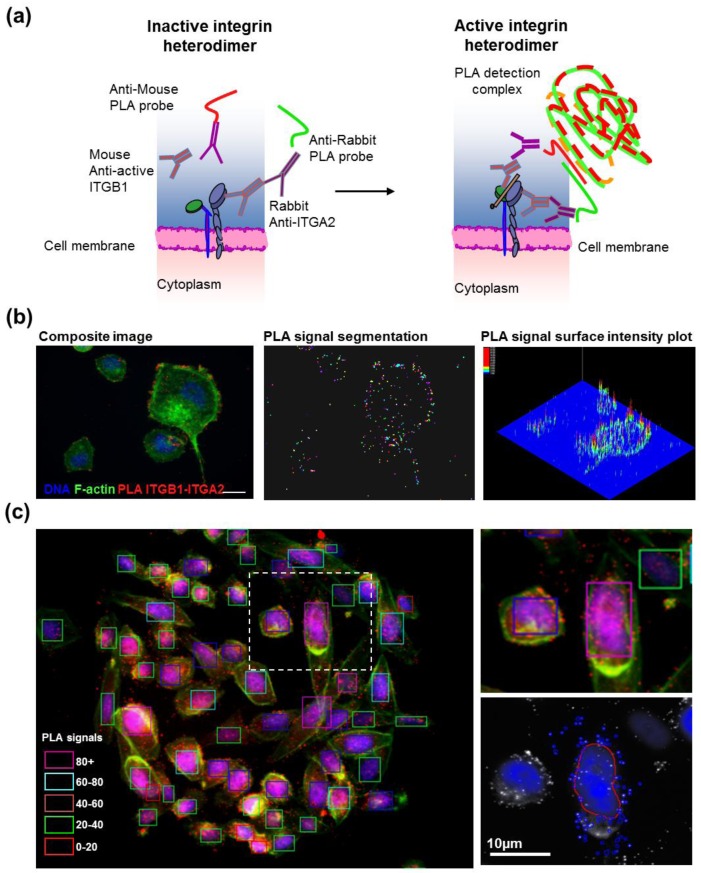
(**a**) Principle of the PLA readout for analysis of active state heterodimeric integrin α2β1 receptors. Active integrin α2β1 heterodimers were recognized in pairs by rabbit monoclonal antibodies binding the alpha subunit ITGA2 and an active conformation-specific mouse monoclonal antibodies binding ITGB1 (clone 12G10). The primary antibodies were bound by species-specific probe antibodies, conjugated to PLA oligonucleotides. When in proximity, the oligonucleotides can be used as templates for the joining of two additional linear oligonucleotides into a DNA circle forming the template for rolling-circle amplification and fluorescence detection of interacting proteins *in situ*. (**b**) Illustration of the use of the PLA technique to assess DNA (blue), F-actin (green) and ITGB1-ITGA2 proximity (red) in individual cells. PLA signals were quantitated at the single signal level using automated image analysis object segmentation algorithms. Scale bar 10 µm. (**c**) CSMA detection of active α2β1-integrin in PC3 prostate cancer cells. Cells on spots were divided into groups according to the amount of PLA signals identified by signal segmentation (right panel) within the membrane boundaries of individual cells detected on basis of F-Actin staining. The colored rectangles surrounding each cell indicate the number of PLA signals associated with the given cell.

Interactions between proteins enable and regulate a wide range of cellular processes. Protein–protein interactions typically are studied using methods such as mass spectrometry of immunoprecipitated protein complexes or via analysis in yeast two hybrid systems. These methods reveal a broad range of interacting proteins. However, they do not reveal where within cells these proteins interact nor do they assess the impact of RNAi knockdown on these interactions. This can only be accomplished using image-based analysis strategies. To this end, we have developed a strategy for *in situ* analysis of protein–protein interactions on cells grown on CSMAs using an antibody-based proximity ligation assay (PLA) [25]. The PLA method detects interacting target proteins in fixed cells by detecting the interactions between paired primary antibodies labeled with complementary oligonucleotides. The oligonucleotides interact to enable rolling circle amplification and oligonucleotide complex formation when the two antibodies are in close proximity (Figure 5(a)). The resulting bright fluorescent complexes can be imaged by standard fluorescence microscopy and the numbers of foci can be measured as a function of location within individual cells (Figure 5(a,b)). To date, PLA analyses has been extensively used to study protein interactions and protein co-localizations in the individual biological experiments and in a small-scale screening of chemical inhibitors of PDGF signaling [26]. The PLA technology can be adapted to high throughput RNAi manipulation of genes that (a) regulate protein–protein interactions; (b) participate in the interactions; or that (c) influence the localization of the interactions. However, it is prohibitively expensive when implemented in multiwell formats, due to the need for large quantities of the detection reagents including enzymes required for the DNA rolling circle amplification (Figure 5(a)). Implementation of PLA analysis in CSMA format allows assessment of the impact of the several thousand RNAi reactions using the same volume of PLA detection reagents that would be required to stain two individual 96-microplate wells [26]. This dramatically reduces reagent consumption, allowing use of PLA for large-scale functional genomics experiments.

Figure 5 illustrates the application of the PLA technique to assessment of detection of activated heterodimeric integrin α2β1 cell-adhesion receptors in cells grown on CSMAs carrying siRNAs. This assay, described previously [10], enables detection of the ITGB1 sub-units with an active conformation in close proximity to ITGA2 alpha sub-units (Figure 5(a)). These results indicate that PLA staining on cell spot microarrays in combination with automated image cytometry can reliably detect PLA signals in single cells (Figure 5(b)) and therefore can be used for functional interrogation of regulators of endogenous protein interactions *in situ*. Analysis of protein proximity after PLA staining is accomplished by segmenting the individual PLA signals and quantifying the number of individual PLA signals per cell. In Figure 5(c), single cells were defined by segmenting according to the extent of F-Actin staining. This analysis enables cells to be classified according to the extent specific protein interactions (Figure 5(c)) or according to the spatial distribution of the protein complexes (Figure 5(b)). We anticipate that assessment of the impact of large-scale siRNA knockdown on specific protein interactions will facilitate elucidation of mechanisms that control protein–protein interactions and/or that regulate their spatial localization. 

### 3.5. Summary

The CSMA analysis platform represents a technological advance in RNAi screening. It enables assessment of the functional impact of large-scale gene knockdown in many different cell types quickly and at low reagent cost. The impact of siRNA knockdown on multiple molecular and cellular response endpoints can be revealed using immunofluorescent staining or fluorescence *in situ* hybridization or a combination of both. Analyses can be carried out for cells growing in diverse culture conditions and during treatment with therapeutic compounds or other siRNAs. Assays for >25 response endpoints already have been developed for the CSMA platform. Automated, image analysis allows images of cells on individual array spots to be acquired and analyzed the amount and spatial distribution of molecular features associated with several cancer hallmarks. We have demonstrated applications of the CSMA technology ranging from general cancer target gene discovery to detailed analyses of specific cellular processes including regulation of integrin cell-adhesion receptors [10,11]. The panel of assays compatible for screening using CSMA can be tailored to address many different biological questions. We expect the number of image cytometry endpoints to expand significantly during the coming years as genomics analyses of cancers and other disease types mature. 

## 4. Future Directions

The ability to assess changes induced by targeted RNAis using image cytometry assays enables assessment of the functional importance of a large number of disease-linked genomic aberrations and aberrant regulatory networks. Loss-of-function genetic screening has been used widely to assess the impact of RNAi knockdown on cell viability and/or immortalization. More recently, imaging-based screening strategies, coupled with immunofluorescent staining for cancer hallmarks has expanded the range of biological endpoints that can be assessed during RNAi screening. The ability to simultaneously assess the levels of specific proteins and RNA species, their spatial locations and the proximities of specific proteins, greatly increases the information that can be gained from RNAi screening experiments. This powerful form of high-throughput gene function assessment at cellular and subcellular resolution has already proven useful for functional annotation of normal and aberrant human genes. Several emerging imaging techniques such as automated confocal microscopy, super-resolution fluorescence microscopy and integrated light and electron microscopy will likely lead to further developments in the field of cell biological gene function assessment by enabling quantitative analysis of RNAi-induced changes in cellular function and structure. Understanding of human genetic and cell biological functions is increasing as the range of biomarkers for specific cellular processes increases. Multiplex analysis on CSMA carrying RNAis allows correlative analyses of the impact of gene perturbations on the molecular and cellular features they regulate in individual cells. In the future, RNAi screening experiments will likely shift from general cell biological discovery with established model cell lines to clinical applications where RNAi screening is performed directly with patient derived cells [6]. 

## Appendix

**Table S1:** Surrogate markers for image cytometry-based quantitative cancer phenotypes.

	**Marker**	**Vendor**	**Cat #**	**Dilution**
**Proliferation**	Ki-67	Abcam	Ab15580	1:400
	CDT1	Cell Signaling Tech.	8064	1:300
	BrdU	BD Pharmingen	555627	1:400
	EdU (Click-iT A647 kit)	Life Technologies	C10356	10 µM
**Apoptosis Senescence**	Cleaved PARP	Cell Signaling Tech.	9546S	1:400
	Histone H3 (K9me^3^)	Abcam	Ab8898	1:300
**DNA Damage**	Gamma-H2Ax	Abcam	Ab11174	1:300
	P53BP1	Novus Biologicals	NB100-904	1:300
	CHEK2 (phospho T68)	Abcam	Ab38461	1:200
	ATM (phospho S1981)	Abcam	Ab36810	1:300
	TP53 (acetyl K381)	Abcam	Ab61241	1:300
**Differentiation**	KRT8	Abcam	Ab59400	1:300
	KRT14	Abcam	Ab7800	1:300
	CTNNB1	Cell Signaling Tech.	8814	1:300
	EpCAM	Cell Signaling Tech.	2929	1:200
	CDH1 (total)	Cell Signaling Tech.	3195	1:200
	CDH1 (extracellular epitope)	Abcam	Ab1416	1:200
	Vimentin	Cell Signaling Tech.	5741	1:300
**Adhesion Matrix**	Active Integrin Beta-1 (12G10)	Abcam	Ab30394	1:200
	Vinculin	Abcam	Ab18058	1:200
	Fibronectin	Epitomics	1574-1	1:300
**Cytoskeleton**	Beta-Tubulin	Santa Cruz Biotech.	Sc-55529	1:300
	F-Actin (phalloidin)	Life Technologies	A12379	1:100
